# Engaging and Recruiting Latino‐led Community Organizations to Build Workforce Capacity in AD/Brain Health Prevention Research

**DOI:** 10.1002/alz.089532

**Published:** 2025-01-09

**Authors:** Mirella Diaz‐Santos

**Affiliations:** ^1^ David Geffen School of Medicine, University of California, Los Angeles, Los Angeles, CA USA

## Abstract

**Background:**

AD prevention and early detection are crucial to reduce prevalence projections in Latinos. Despite these projections, our community partners in Los Angeles County continue to disclose barriers in accessing culturally and linguistically congruent bilingual programs activating behavioral modifications for AD prevention and early identification of cognitive changes. In this study, we partnered with community organizations to co‐design and pilot a brain health prevention program tailored to the needs of their constituents.

**Method:**

We used a mixed‐methods design using quantitative data from surveys, and qualitative data from informal short interviews in Spanish and English with Latino community organizations and their constituents. We implemented Community‐Based Participatory Research (CBPR) and Human Centered Design (HCD) principles, anchoring the program in the cognitive reserve theory, tailored to cultural values, traditions, and needs of Latinos in Los Angeles County. Spanish and English‐speaking Latino members requested brain health and early detections materials easy to comprehend. Four Spanish and English brochures were designed. We also design a communication training toolkit to improve delivery of the program from our research team, employing oral storytelling techniques with cultural metaphors explaining brain structure/functions and cognitive functioning/decline.

**Results:**

Between June and December 2023, our team received invitations to participate in 11 community engagement and outreach events across Los Angeles County. A team of 3 bilingual trained research assistants engaged with approximately 475 Spanish and English‐speaking community members with an average of 43 individuals per event. Average distance traveled averaged 37 miles roundtrip total per event. Total printing cost per event was approximately $292, with an additional $30 for giveaways. In the 6 months of implementation, a total of thirty community‐based organizations requested formal presentations and trainings for their staff. Overarching qualitative themes included the community‐centeredness of the program, it’s cultural, linguistically and health literacy appropriateness, and the culturally competent communication skills showcased by bilingual research staff.

**Conclusion:**

Following CBPR, and HCD principles, we showed respect for community needs, while empowering the members to be an integral part of the design and pilot. Our approach engaged and recruited 30 Latino‐led community organizations interested in building their own workforce capacity in brain health prevention and early detection.

